# Supplementary material for: A Likelihood Approach for Real-Time Calibration of Stochastic Compartmental Epidemic Models
Source: PLoS Comput Biol. 2017 Jan 17;13(1):e1005257. doi: 10.1371/journal.pcbi.1005257 (PMC5240920; doi:10.1371/journal.pcbi.1005257)
Supplement: S1 File — (TAR.GZ) [file pcbi.1005257.s014.tar.gz › HSPH_Online-SI-Revision/IPoi09/n100-extreme/n100-extreme_BENCH2_table4.pdf]

1	newly 3 weeks spec	2392.	2393.74	{1115., 4287.}	45.1235	0.182429	newly 3 weeks cumul	9902.	10 006.4	{5086., 18 145.}	40.3499	0.164143	newly 5 weeks spec	1155.	1228.21	{565., 2281.}	47.626	0.177284	newly 5 weeks cumul	12 790.	12 957.2	{6507., 23 543.}	41.7936	0.16836	newly 50 weeks cumul	15 830.	15 913.8	{7845., 28 889.}	43.3065	0.174882
2	newly 3 weeks spec	2435.	1414.28	{694., 2424.}	57.5528	0.360406	newly 3 weeks cumul	9225.	5626.92	{2835., 9699.}	54.9059	0.333578	newly 5 weeks spec	1337.	834.361	{400., 1433.}	56.84	0.34109	newly 5 weeks cumul	12 421.	7551.13	{3763., 12 928.}	55.6018	0.338518	newly 46 weeks cumul	16 331.	10 134.4	{4942., 17 440.}	55.9374	0.336497
3	newly 3 weeks spec	1444.	1990.37	{932., 3609.}	56.3812	0.167568	newly 3 weeks cumul	6755.	8605.94	{4284., 15 262.}	49.4272	0.160252	newly 5 weeks spec	609.	988.839	{463., 1749.}	73.7804	0.194061	newly 5 weeks cumul	8307.	11 002.9	{5377., 19 530.}	52.5233	0.163641	newly 51 weeks cumul	9551.	13 263.	{6345., 23 700.}	56.508	0.168402
4	newly 3 weeks spec	766.	1529.64	{764., 2766.}	102.303	0.246606	newly 3 weeks cumul	4576.	8098.79	{4244., 14 428.}	81.7616	0.211499	newly 5 weeks spec	254.	549.803	{265., 1007.}	116.755	0.267778	newly 5 weeks cumul	5299.	9563.43	{4991., 17 015.}	84.8555	0.216726	newly 55 weeks cumul	5634.	10 370.2	{5413., 18 617.}	88.0907	0.222741
5	newly 3 weeks spec	1978.	2790.27	{1386., 4886.}	57.17	0.169067	newly 3 weeks cumul	9313.	11 788.1	{6176., 20 608.}	46.3738	0.153604	newly 5 weeks spec	894.	1408.15	{643., 2519.}	70.5835	0.189922	newly 5 weeks cumul	11 542.	15 186.7	{7751., 26 736.}	49.897	0.15842	newly 52 weeks cumul	13 327.	18 435.6	{9169., 32 655.}	54.5479	0.165151
6	newly 3 weeks spec	2370.	2120.26	{1032., 3850.}	45.441	0.212087	newly 3 weeks cumul	9890.	8787.26	{4372., 16 139.}	42.7769	0.201787	newly 5 weeks spec	1132.	1127.92	{565., 2167.}	46.9487	0.196206	newly 5 weeks cumul	12 727.	11 461.9	{5710., 21 147.}	43.5785	0.202618	newly 50 weeks cumul	15 600.	14 350.9	{7169., 26 425.}	44.1992	0.20112
7	newly 3 weeks spec	1951.	3614.77	{1792., 6518.}	88.3484	0.225798	newly 3 weeks cumul	8547.	13 744.6	{7392., 24 420.}	66.2392	0.190524	newly 5 weeks spec	968.	2168.58	{1013., 3905.}	125.327	0.280817	newly 5 weeks cumul	10 776.	18 737.5	{9741., 33 236.}	77.7606	0.210164	newly 48 weeks cumul	12 929.	25 283.5	{12 530., 44 960.}	97.6657	0.243676
8	newly 3 weeks spec	2287.	2857.	{1395., 5135.}	48.161	0.160236	newly 3 weeks cumul	9859.	11 625.9	{6047., 21 201.}	42.2683	0.150772	newly 5 weeks spec	1178.	1546.7	{763., 2841.}	53.0699	0.165763	newly 5 weeks cumul	12 762.	15 292.9	{7809., 27 892.}	44.1281	0.15398	newly 50 weeks cumul	15 717.	19 309.8	{9837., 35 337.}	46.3583	0.156899
9	newly 3 weeks spec	2292.	3810.	{2008., 7154.}	71.4474	0.194352	newly 3 weeks cumul	10 287.	15 044.5	{8533., 26 935.}	54.12	0.165142	newly 5 weeks spec	1160.	2120.26	{1032., 3850.}	87.4194	0.220254	newly 5 weeks cumul	13 098.	20 021.8	{10 897., 35 920.}	59.972	0.1755	newly 50 weeks cumul	15 554.	25 585.4	{13 583., 46 206.}	69.8579	0.193394
10	newly 3 weeks spec	296.	1514.08	{798., 2846.}	411.514	0.654538	newly 3 weeks cumul	1966.	7717.21	{4136., 14 687.}	292.534	0.544266	newly 5 weeks spec	92.	586.148	{297., 1128.}	537.117	0.746267	newly 5 weeks cumul	2237.	9243.59	{4904., 17 744.}	313.214	0.565393	newly 54 weeks cumul	2337.	10 182.3	{5411., 19 615.}	335.699	0.587861
11	newly 3 weeks spec	2110.	2824.27	{1374., 4975.}	53.8717	0.164987	newly 3 weeks cumul	9055.	10 984.	{5542., 19 605.}	45.3033	0.156132	newly 5 weeks spec	1137.	1649.55	{724., 2931.}	63.3699	0.177469	newly 5 weeks cumul	11 827.	14 802.1	{7209., 26 369.}	48.4211	0.160302	newly 48 weeks cumul	14 734.	19 698.5	{9320., 35 131.}	53.6463	0.164354
12	newly 3 weeks spec	2018.	3296.2	{1631., 5855.}	70.9987	0.194588	newly 3 weeks cumul	9115.	13 019.9	{6877., 22 875.}	53.33	0.164524	newly 5 weeks spec	959.	1849.28	{886., 3385.}	97.4359	0.236814	newly 5 weeks cumul	11 517.	17 349.5	{9020., 30 864.}	59.903	0.1757	newly 49 weeks cumul	13 641.	22 342.4	{11 249., 39 833.}	71.0223	0.195399
13	newly 3 weeks spec	3118.	2371.75	{1221., 4091.}	47.9926	0.25274	newly 3 weeks cumul	12 156.	9152.95	{4878., 15 768.}	44.7273	0.239132	newly 5 weeks spec	2008.	1414.28	{694., 2424.}	53.3889	0.295824	newly 5 weeks cumul	16 586.	12 408.1	{6534., 21 376.}	46.4718	0.248725	newly 47 weeks cumul	22 880.	16 915.6	{8742., 29 117.}	48.7049	0.262039
14	newly 3 weeks spec	249.	3825.29	{1984., 6767.}	1436.26	1.12974	newly 3 weeks cumul	1934.	15 694.6	{8675., 26 901.}	711.508	0.867901	newly 5 weeks spec	37.	1990.37	{932., 3609.}	5279.38	1.66051	newly 5 weeks cumul	2082.	20 475.2	{10 975., 35 396.}	883.439	0.94598	newly 52 weeks cumul	2131.	25 132.3	{13 036., 43 834.}	1079.37	1.01988
15	newly 3 weeks spec	658.	2244.11	{1211., 4220.}	241.051	0.472866	newly 3 weeks cumul	3705.	10 131.3	{5713., 18 678.}	173.45	0.384798	newly 5 weeks spec	197.	1036.98	{541., 1946.}	426.388	0.657055	newly 5 weeks cumul	4246.	12 698.9	{7105., 23 560.}	199.079	0.421474	newly 52 weeks cumul	4525.	14 810.1	{8240., 27 498.}	227.296	0.458862
16	newly 3 weeks spec	902.	1647.7	{740., 2561.}	90.651	0.226549	newly 3 weeks cumul	5020.	7577.38	{3526., 11 924.}	64.9703	0.183284	newly 5 weeks spec	342.	739.279	{324., 1139.}	119.701	0.27016	newly 5 weeks cumul	5877.	9434.11	{4349., 14 712.}	72.1055	0.194288	newly 52 weeks cumul	6347.	10 911.1	{5012., 16 933.}	80.9981	0.208818
17	newly 3 weeks spec	2633.	3768.23	{2085., 6516.}	54.7807	0.165806	newly 3 weeks cumul	10 424.	13 957.7	{8450., 23 409.}	45.0927	0.149014	newly 5 weeks spec	1461.	2371.75	{1221., 4091.}	71.2604	0.188114	newly 5 weeks cumul	13 884.	19 342.4	{11 285., 32 661.}	50.2121	0.157901	newly 47 weeks cumul	18 108.	27 105.1	{15 043., 46 010.}	59.0245	0.171329
18	newly 3 weeks spec	1965.	3952.47	{1992., 7191.}	102.334	0.258291	newly 3 weeks cumul	8760.	14 759.7	{8092., 25 834.}	71.6915	0.205947	newly 5 weeks spec	847.	2425.32	{1130., 4177.}	186.343	0.387276	newly 5 weeks cumul	10 903.	20 303.1	{10 702., 35 448.}	87.9656	0.235714	newly 48 weeks cumul	12 657.	27 887.9	{13 963., 48 359.}	120.639	0.292485
19	newly 3 weeks spec	2260.	3810.	{2008., 7154.}	73.2975	0.197969	newly 3 weeks cumul	9867.	15 044.5	{8533., 26 935.}	58.6736	0.174715	newly 5 weeks spec	1026.	2120.26	{1032., 3850.}	108.718	0.260626	newly 5 weeks cumul	12 362.	20 021.8	{10 897., 35 920.}	66.9891	0.19005	newly 50 weeks cumul	14 680.	25 585.4	{13 583., 46 206.}	78.0259	0.210321
20	newly 3 weeks spec	351.	1529.64	{764., 2766.}	335.795	0.573158	newly 3 weeks cumul	2657.	8098.79	{4244., 14 428.}	204.809	0.424172	newly 5 weeks spec	80.	549.803	{265., 1007.}	587.254	0.768199	newly 5 weeks cumul	2903.	9563.43	{4991., 17 015.}	229.433	0.456782	newly 55 weeks cumul	3010.	10 370.2	{5413., 18 617.}	244.526	0.475527
21	newly 3 weeks spec	1266.	2422.57	{1124., 3822.}	96.417	0.234828	newly 3 weeks cumul	6452.	10 858.8	{5342., 16 918.}	75.9073	0.204208	newly 5 weeks spec	504.	1117.46	{495., 1703.}	124.398	0.279343	newly 5 weeks cumul	7741.	13 624.	{6576., 21 182.}	82.5161	0.214853	newly 52 weeks cumul	8578.	15 840.2	{7541., 24 488.}	89.9222	0.226777
22	newly 3 weeks spec	1097.	1660.37	{907., 2701.}	60.5449	0.16814	newly 3 weeks cumul	5662.	7966.92	{4375., 12 777.}	52.3464	0.15526	newly 5 weeks spec	414.	690.968	{350., 1087.}	73.352	0.191727	newly 5 weeks cumul	6768.	9737.81	{5322., 15 571.}	54.6523	0.158779	newly 53 weeks cumul	7400.	10 983.4	{6028., 17 567.}	57.7842	0.163762
23	newly 3 weeks spec	2274.	2371.75	{1221., 4091.}	44.2342	0.17473	newly 3 weeks cumul	9195.	9152.95	{4878., 15 768.}	40.2555	0.169209	newly 5 weeks spec	1192.	1414.28	{694., 2424.}	50.6615	0.171844	newly 5 weeks cumul	12 094.	12 408.1	{6534., 21 376.}	42.1272	0.170522	newly 46 weeks cumul	15 344.	16 915.6	{8742., 29 117.}	45.049	0.167598
24	newly 3 weeks spec	2818.	2969.5	{1686., 4617.}	39.5517	0.155805	newly 3 weeks cumul	11 319.	11 134.	{6571., 17 214.}	33.6562	0.146176	newly 5 weeks spec	1477.	1868.	{954., 2787.}	51.0607	0.161011	newly 5 weeks cumul	14 831.	15 388.6	{8821., 23 733.}	36.74	0.148945	newly 46 weeks cumul	19 249.	21 577.9	{11 839., 32 805.}	41.9865	0.154073
25	newly 3 weeks spec	1110.	3296.2	{1631., 5855.}	196.955	0.416177	newly 3 weeks cumul	5663.	13 019.9	{6877., 22 875.}	130.243	0.319326	newly 5 weeks spec	432.	1849.28	{886., 3385.}	328.074	0.560665	newly 5 weeks cumul	6746.	17 349.5	{9020., 30 864.}	157.243	0.361411	newly 49 weeks cumul	7332.	22 342.4	{11 249., 39 833.}	204.725	0.42896
26	newly 3 weeks spec	2919.	3012.97	{1620., 5161.}	41.3121	0.166669	newly 3 weeks cumul	11 387.	11 397.8	{6459., 19 421.}	36.3584	0.154232	newly 5 weeks spec	1758.	1840.89	{920., 3184.}	46.2653	0.180802	newly 5 weeks cumul	15 461.	15 610.4	{8611., 26 696.}	38.6132	0.160823	newly 47 weeks cumul	21 236.	21 532.2	{11 480., 36 861.}	41.5329	0.170465
27	newly 3 weeks spec	2411.	1361.46	{614., 2526.}	56.574	0.363302	newly 3 weeks cumul	9503.	5691.03	{2711., 10 414.}	54.0616	0.335067	newly 5 weeks spec	1418.	728.246	{333., 1315.}	59.5401	0.401131	newly 5 weeks cumul	12 786.	7419.9	{3462., 13 540.}	55.0577	0.347665	newly 48 weeks cumul	17 179.	9322.52	{4372., 16 958.}	56.8175	0.372541
28	newly 3 weeks spec	2066.	1530.61	{802., 2936.}	48.8977	0.262597	newly 3 weeks cumul	9289.	7028.31	{3862., 13 159.}	46.5276	0.246415	newly 5 weeks spec	1123.	694.145	{368., 1348.}	53.3062	0.321616	newly 5 weeks cumul	12 043.	8759.44	{4787., 16 351.}	47.8488	0.260406	newly 52 weeks cumul	14 773.	10 176.5	{5531., 19 043.}	49.5656	0.27975
29	newly 3 weeks spec	2511.	3934.25	{2155., 6135.}	63.8163	0.185599	newly 3 weeks cumul	10 166.	14 378.5	{8469., 22 198.}	49.3619	0.158632	newly 5 weeks spec	1397.	2519.92	{1223., 3964.}	85.4102	0.218197	newly 5 weeks cumul	13 376.	20 073.7	{11 377., 31 243.}	57.0546	0.173062	newly 46 weeks cumul	17 318.	28 557.5	{15 152., 44 737.}	70.4262	0.196503
30	newly 3 weeks spec	1594.	914.836	{461., 1580.}	54.1014	0.344103	newly 3 weeks cumul	7832.	4955.9	{2498., 8915.}	50.98	0.307115	newly 5 weeks spec	651.	323.574	{167., 653.}	57.9764	0.399806	newly 5 weeks cumul	9505.	5829.28	{2928., 10 575.}	51.9842	0.31885	newly 55 weeks cumul	10 576.	6312.52	{3165., 11 524.}	52.7458	0.328589
31	newly 3 weeks spec	771.	859.694	{440., 1346.}	41.607	0.148515	newly 3 weeks cumul	4574.	5131.4	{2753., 8287.}	40.952	0.145646	newly 5 weeks spec	189.	261.129	{139., 396.}	53.7634	0.155296	newly 5 weeks cumul	5139.	5867.1	{3138., 9344.}	41.6574	0.14526	newly 56 weeks cumul	5392.	6193.19	{3311., 9790.}	41.9181	0.145055
32	newly 3 weeks spec	858.	1908.73	{928., 2812.}	123.197	0.290739	newly 3 weeks cumul	4649.	9185.62	{4820., 13 310.}	99.6806	0.253283	newly 5 weeks spec	288.	792.583	{416., 1161.}	175.434	0.37647	newly 5 weeks cumul	5438.	11 209.4	{5897., 16 273.}	107.784	0.267677	newly 53 weeks cumul	5832.	12 600.7	{6608., 18 278.}	117.156	0.284119
33	newly 3 weeks spec	3068.	1990.37	{932., 3609.}	52.8972	0.312234	newly 3 weeks cumul	12 287.	8605.94	{4284., 15 262.}	48.7993	0.274996	newly 5 weeks spec	1670.	988.839	{463., 1749.}	55.8451	0.349328	newly 5 weeks cumul	16 122.	11 002.9	{5377., 19 530.}	50.1368	0.2873	newly 51 weeks cumul	20 713.	13 263.	{6345., 23 700.}	52.1736	0.311156
34	newly 3 weeks spec	2614.	3175.34	{1631., 5081.}	46.2666	0.16114	newly 3 weeks cumul	10 179.	11 889.9	{6617., 18 631.}	40.2813	0.148496	newly 5 weeks spec	1528.	1972.13	{927., 3181.}	53.8645	0.174171	newly 5 weeks cumul	13 712.	16 381.9	{8709., 25 776.}	43.2726	0.154563	newly 46 weeks cumul	18 212.	22 893.5	{11 627., 36 089.}	48.0849	0.161019
35	newly 3 weeks spec	2810.	2393.74	{1115., 4287.}	47.3378	0.224206	newly 3 weeks cumul	11 877.	10 006.4	{5086., 18 145.}	42.7378	0.207043	newly 5 weeks spec	1428.	1228.21	{565., 2281.}	49.9492	0.231985	newly 5 weeks cumul	15 254.	12 957.2	{6507., 23 543.}	44.1243	0.211117	newly 51 weeks cumul	18 586.	15 913.8	{7845., 28 889.}	45.5006	0.215187
36	newly 3 weeks spec	2377.	1364.73	{647., 2526.}	55.7568	0.352912	newly 3 weeks cumul	9678.	5674.	{2744., 10 283.}	53.7794	0.336539	newly 5 weeks spec	1295.	739.032	{351., 1319.}	57.4094	0.3641	newly 5 weeks cumul	12 751.	7427.18	{3573., 13 440.}	54.5231	0.342315	newly 49 weeks cumul	16 300.	9410.9	{4479., 16 956.}	55.2897	0.348835
37	newly 3 weeks spec	918.	1514.08	{798., 2846.}	71.2043	0.193348	newly 3 weeks cumul	5030.	7717.21	{4136., 14 687.}	61.9933	0.178133	newly 5 weeks spec	306.	586.148	{297., 1128.}	94.2194	0.236802	newly 5 weeks cumul	5864.	9243.59	{4904., 17 744.}	65.1994	0.183663	newly 54 weeks cumul	6382.	10 182.3	{5411., 19 615.}	66.6567	0.186042
38	newly 3 weeks spec	286.	1514.08	{798., 2846.}	429.399	0.669464	newly 3 weeks cumul	2079.	7717.21	{4136., 14 687.}	271.198	0.519995	newly 5 weeks spec	76.	586.148	{297., 1128.}	671.247	0.829241	newly 5 weeks cumul	2297.	9243.59	{4904., 17 744.}	302.42	0.553898	newly 54 weeks cumul	2347.	10 182.3	{5411., 19 615.}	333.843	0.586007
39	newly 3 weeks spec	1569.	3614.77	{1792., 6518.}	130.562	0.306722	newly 3 weeks cumul	6961.	13 744.6	{7392., 24 420.}	98.1888	0.256989	newly 5 weeks spec	719.	2168.58	{1013., 3905.}	201.611	0.403846	newly 5 weeks cumul	8738.	18 737.5	{9741., 33 236.}	114.806	0.284428	newly 48 weeks cumul	10 021.	25 283.5	{12 530., 44 960.}	152.305	0.344449
40	newly 3 weeks spec	2069.	2120.26	{1032., 3850.}	45.0103	0.183604	newly 3 weeks cumul	8421.	8787.26	{4372., 16 139.}	42.5038	0.170974	newly 5 weeks spec	1045.	1127.92	{565., 2167.}	47.3386	0.181591	newly 5 weeks cumul	10 908.	11 461.9	{5710., 21 147.}	43.49	0.173223	newly 49 weeks cumul	13 560.	14 350.9	{7169., 26 425.}	44.3912	0.175127
41	newly 3 weeks spec	1243.	2393.74	{1115., 4287.}	96.2033	0.231737	newly 3 weeks cumul	5945.	10 006.4	{5086., 18 145.}	73.8045	0.196632	newly 5 weeks spec	608.	1228.21	{565., 2281.}	104.985	0.240382	newly 5 weeks cumul	7464.	12 957.2	{6507., 23 543.}	78.7714	0.203982	newly 50 weeks cumul	8541.	15 913.8	{7845., 28 889.}	90.0012	0.222768
42	newly 3 weeks spec	457.	940.23	{470., 1832.}	106.966	0.262086	newly 3 weeks cumul	2671.	4867.39	{2563., 9483.}	85.2447	0.221709	newly 5 weeks spec	82.	359.738	{187., 685.}	338.705	0.584909	newly 5 weeks cumul	2987.	5813.28	{3029., 11 346.}	96.2515	0.243031	newly 54 weeks cumul	3092.	6392.25	{3377., 12 482.}	107.441	0.264643
43	newly 3 weeks spec	2726.	3768.23	{2085., 6516.}	51.9118	0.161385	newly 3 weeks cumul	11 008.	13 957.7	{8450., 23 409.}	41.2713	0.142628	newly 5 weeks spec	1377.	2371.75	{1221., 4091.}	78.2599	0.199322	newly 5 weeks cumul	14 359.	19 342.4	{11 285., 32 661.}	47.5887	0.153648	newly 47 weeks cumul	18 000.	27 105.1	{15 043., 46 010.}	59.5791	0.172198
44	newly 3 weeks spec	1026.	913.2	{425., 1344.}	42.4821	0.199683	newly 3 weeks cumul	5335.	4651.08	{2138., 7049.}	41.7098	0.200323	newly 5 weeks spec	393.	364.55	{165., 557.}	42.3113	0.191985	newly 5 weeks cumul	6350.	5595.55	{2573., 8517.}	41.7661	0.198584	newly 53 weeks cumul	7041.	6194.3	{2816., 9422.}	41.9368	0.199715
45	newly 3 weeks spec	374.	1705.23	{895., 2739.}	355.943	0.601998	newly 3 weeks cumul	2546.	7930.26	{4350., 13 190.}	211.479	0.442043	newly 5 weeks spec	135.	754.726	{390., 1262.}	459.056	0.685404	newly 5 weeks cumul	2913.	9828.94	{5263., 16 399.}	237.416	0.475033	newly 52 weeks cumul	3058.	11 301.1	{6002., 18 710.}	269.56	0.513332
46	newly 3 weeks spec	1322.	3825.29	{1984., 6767.}	189.356	0.404711	newly 3 weeks cumul	7028.	15 694.6	{8675., 26 901.}	123.641	0.30902	newly 5 weeks spec	520.	1990.37	{932., 3609.}	282.764	0.512713	newly 5 weeks cumul	8416.	20 475.2	{10 975., 35 396.}	143.448	0.340063	newly 52 weeks cumul	9218.	25 132.3	{13 036., 43 834.}	172.644	0.383831
47	newly 3 weeks spec	877.	1514.08	{798., 2846.}	77.3202	0.205185	newly 3 weeks cumul	5116.	7717.21	{4136., 14 687.}	60.2674	0.175138	newly 5 weeks spec	280.	586.148	{297., 1128.}	110.31	0.267354	newly 5 weeks cumul	5861.	9243.59	{4904., 17 744.}	65.2555	0.183761	newly 54 weeks cumul	6385.	10 182.3	{5411., 19 615.}	66.6046	0.185952
48	newly 3 weeks spec	1336.	2283.23	{1086., 3469.}	77.3752	0.209038	newly 3 weeks cumul	7014.	11 119.9	{5514., 16 839.}	65.5411	0.187545	newly 5 weeks spec	498.	913.2	{425., 1344.}	88.4873	0.227943	newly 5 weeks cumul	8264.	13 487.7	{6566., 20 419.}	69.7564	0.195836	newly 54 weeks cumul	8948.	15 031.	{7244., 22 792.}	73.9985	0.204255
49	newly 3 weeks spec	215.	859.694	{440., 1346.}	299.857	0.539553	newly 3 weeks cumul	1583.	5131.4	{2753., 8287.}	224.157	0.451839	newly 5 weeks spec	43.	261.129	{139., 396.}	507.277	0.718207	newly 5 weeks cumul	1735.	5867.1	{3138., 9344.}	238.161	0.469665	newly 56 weeks cumul	1768.	6193.19	{3311., 9790.}	250.294	0.484822
50	newly 3 weeks spec	2185.	3118.06	{1485., 5437.}	57.6556	0.170854	newly 3 weeks cumul	9176.	11 948.8	{6083., 21 264.}	47.311	0.155185	newly 5 weeks spec	1236.	1859.42	{841., 3195.}	65.2156	0.179067	newly 5 weeks cumul	12 158.	16 233.6	{8024., 28 636.}	50.6081	0.16029	newly 47 weeks cumul	15 907.	21 959.	{10 452., 38 352.}	55.0474	0.166897
